# Water-Supply and Stream-Pollution in Illinois

**Published:** 1889-04

**Authors:** 


### WATER-SUPPLY AND STREAM-POLLUTION IN ILLINOIS.

The Secretary of the Illinois State Board of Health has recently issued a preliminary report on the "Water Supplies of Illinois and the Pollution of its Streams." This investigation and study were among the earliest efforts of the State Board of Health, though they were at first necessarily confined to pressing emergencies. Within the past twelve months the work has been pushed with great vigor and on a broad scale, the chemical work being done by Professor John H. Long, and the engineering work and estimates by Mr. L. E. Cooley, whose report on "The Illinois River Basin in Its Relation to Sanitary Engineering" is both interesting and exhaustive.

The work of the Board in this connection is by no means purely local, nor is it confined to the valley of the Illinois river; it covers already 3,500 miles of water-courses—all the streams in the State except the Rock river in the northern, and the tributaries of the Wabash and Ohio, and of the Mississippi below East St. Louis, as well as the water-supplies of all the State Institutions, and of the majority of the important cities and towns. The data secured embrace over 1,000 chemical analyses of various waters under different conditions, the meteorological conditions for a series of years, the physical conditions relating to questions of sanitary engineering, and other factors of the very complicated problem. No such work on so large a scale has been attempted before in this country.

Of the many important fields of work that must be covered by an efficient board of health, none is more important than those of water-supply and drainage. In this preliminary report are discussed the general physical characteristics of the Illinois and Lake Michigan basins, with the general effect of inhabitation, and a part of the principal tributary basins; also a partial report of the distribution and changes in population in the two basins. Great as is the amount of work shown in the report, still the report is but preliminary.

The effects of inhabitation have already been shown on many of the water-courses of the State, and it is certain that the next half-century will bring about the most radical alteration in the flow of the streams.

If left to themselves these streams will ultimately convert no inconsiderable part of the State into marshes. Some of the streams will run dry at certain seasons of the year, while at other seasons the floods will be increased. A considerable proportion of these effects will occur within a few years as the result of wholesale reclamation projects. They will be accompanied by an enormous increase in the amount of detritus, which will not keep up indefinitely in the future, but will be at all times multiplied over past conditions, owing to the increased tillage, and the more complete ditching of the lands. "The ultimate effect of the changed conditions in the lower valley of the Illinois will be to convert it into a continuous marsh, without well-defined drainage-lines other than a chain of sloughs, except, perhaps, at the lower end, gradually filling up its bottoms until in ages of time it may probably become adjusted to its new labors. It is doubtful if man can counteract these tendencies or more than postpone their consummation without a radical change in conditions." It must be the work of art to change these conditions.

The question of the dilution and disposal of Chicago sewage is one of great interest to a large portion of the State. More study will be required to determine with accuracy the limit necessary for the effective dilution of sewage, but from what is now known it may be safely said that the limit is within the practical means of accomplishment. This matter is intimately connected with that of distribution of population. The urban population of Chicago is about equal to that of the remainder of the State, double that on the Illinois river watershed, and many times that of the Illinois valley. These are facts of the greatest importance in considering the necessities of Chicago. And certainly, if the solution of the sanitary necessities of one of the largest and most important cities of the country can be made of benefit to many other important communities, and of vast importance to the commercial, economic, and sanitary interests of a large portion of Illinois, there can be no question as to a wise State policy on the subject. And in this connection it may be remarked that it is an unfortunate characteristic of American legislative bodies to do things in too much haste, and without due consideration of the whole matter. Thus, plans are entered into, and hundreds of thousands of dollars spent in projects that have been tried and found to be failures in the old world. Hundreds of thousands of dollars, and a great deal of valuable time, could be saved by sending commissioners to Europe to study the sanitary methods and systems of such countries as England, Germany and France.

It is impossible in any ordinary amount of space to give even an analysis of the preliminary report, on account of its very condensed nature. We can say, however, that the work thus far completed is of paramount importance, and is a severe criticism of the recent action of the lower House of the Illinois Legislature in refusing to make appropriations for the State Board of Health. For the work that has been done great credit is due the Secretary of the Board, to Professor Long, and to Engineer Cooley. To complete this most important work the Board must have funds, and for the sake of the health of the people of the State it is to be hoped that the Board will not be hampered in this matter by unwise legislation.

